# Spatiotopic Coding of BOLD Signal in Human Visual Cortex Depends on Spatial Attention

**DOI:** 10.1371/journal.pone.0021661

**Published:** 2011-07-07

**Authors:** Sofia Crespi, Laura Biagi, Giovanni d'Avossa, David C. Burr, Michela Tosetti, Maria Concetta Morrone

**Affiliations:** 1 Department of Psychology, Università Degli Studi di Firenze, Florence, Italy; 2 Department of Psychology, Università Vita-Salute San Raffaele, Milan, Italy; 3 Fondazione Stella Maris, Calambrone, Pisa, Italy; 4 School of Psychology Adeilad Brigantia, Bangor University, Bangor, United Kingdom; 5 Istituto di Neuroscienze, CNR, Pisa, Italy; 6 Department of Physiological Sciences, University of Pisa, Pisa, Italy; 7 Department of Robotic, Brain and Cognitive Sciences, Istituto Italiano di Tecnologia, Genova, Italy; The University of Melbourne, Australia

## Abstract

The neural substrate of the phenomenological experience of a stable visual world remains obscure. One possible mechanism would be to construct *spatiotopic* neural maps where the response is selective to the position of the stimulus in external space, rather than to retinal eccentricities, but evidence for these maps has been inconsistent. Here we show, with fMRI, that when human subjects perform concomitantly a demanding attentive task on stimuli displayed at the fovea, BOLD responses evoked by moving stimuli irrelevant to the task were mostly tuned in retinotopic coordinates. However, under more unconstrained conditions, where subjects could attend easily to the motion stimuli, BOLD responses were tuned not in retinal but in external coordinates (spatiotopic selectivity) in many visual areas, including MT, MST, LO and V6, agreeing with our previous fMRI study. These results indicate that spatial attention may play an important role in mediating spatiotopic selectivity.

## Introduction

The world appears stable and unchanging despite the continuous spatial transformations imposed by eye, head and body movements, suggesting that there may exist in our brain a spatial representation encoded not in retinal, but in craniotopic or spatiotopic coordinates, that encodes location independently of where the eyes are looking. The construction of maps of this sort is a non-trivial process, involving combination of retinal-based signals with information about eye position [1], [2]. However, whether such a neural map is explicitly represented in the brain remains a contentious issue. In a landmark study, Andersen and Mountcastle [3] showed that the excitability (or *gain fields*) of cells in the parietal cortex of macaque monkeys depend on gaze. This observation has been verified and extended to much of visual cortex [4], [5], [6]. A series of studies also demonstrated in many visual areas, including V6, VIP and MST, neuronal spatiotopic selectivity in external or craniotopic, rather than retinal coordinates [7], [8], [9], [10], [11]. However, in all cases only a small proportion of neurons showed spatiotopic selectivity, and not all studies have reported effects of this type (see discussion).

Similarly, several fMRI experiments have demonstrated the effect of gaze on modulation of responses in many human cortical areas [12], [13], [14], [15], [16], [17]. d'Avossa et al. [18] showed that gaze modulated the response of area MT+ (the presumed homologous region of monkey MT/MST in humans), creating a strong response selective to retinally ipsilateral stimuli presented in the centre of the screen, while gaze was directed contralaterally. They further showed that the modulation of response created spatiotopic selectivity in screen coordinates, with tuning invariant for gaze shifts (while V1 was clearly retinotopically tuned).

This study has been challenged by Gardner et al. [19] who reported visually evoked BOLD responses in hMT+, along with the rest of human occipital cortex, to be retinotopically rather than spatiotopically selective. One difference in experimental procedure introduced by Gardner et al. [19] was that they required subjects to perform a demanding task on stimuli centered at the fixation point (which moved in external space from fixation to fixation), while we [18] either directed attention to the moving stimulus (with a discrimination task), or allowed subjects to direct attention at will (no competing foveal task) in a less constrained viewing condition and in a companion experiment where only the straight ahead position was measured to allocate full sustained attention to the motion stimuli.

Attention is known to modulate BOLD responses in many areas, including V1 and associative cortex, particularly along the dorsal pathway [20], [21], [22], [23], [24]. Directing attention to the fovea boosts the response to stimuli near the attended target, while suppressing that to irrelevant stimuli distant from the attended location. The effect of attention can even reshape and shift the receptive fields of single cells in monkey MT [25], in BOLD responses of human MT [26] and, to a lesser extent, in human V1 [27]. Attention seems to be allocated both in retinal and spatiotopic coordinates [28], [29], [30], [31], and seems to serve a fundamental role in mediating spatial stability and transfer of information across saccades [32], [33].

The purpose of the current study is therefore to test specifically whether a foveal attentional task can influence the tuning of visually evoked BOLD responses by repeating our test for spatiotopy with and without a demanding central attentive task. Under passive viewing conditions, where attention was free to be directed to the only stimulus visible on the screen, we replicate our previous results, finding clear evidence for spatiotopy in area MT, as well as areas MST, LO and V6. However, when attention was directed to the fovea with a continuous and demanding visual discrimination task, the tuning of the same areas was retinotopic rather than spatiotopic. These results help resolve the controversial issue, and also points to the fundamental role of attention in constructing spatiotopic representations, although the neural mechanism of the modulation remain to be still discovered.

## Materials and Methods

### Subjects

Three healthy adults (two females, one male) took part in the new study and 3 others were reanalyzed from the previous experiment [18]. All subjects were experienced in psychophysical and eye-movement studies and had corrected-to-normal vision. Each subject gave informed consent prior to participation, in accordance with the guidelines of the Human Studies Review Board of the Stella Maris Scientific Institute. Each subject was scanned for a total of over 6 hours in the various conditions, over several days (3 hours for the major experiment (25 6-minute scans), both for the new and old data sets, as well as 2 hours for motion selectivity and retinotopy, and about 1 hour of anatomical scans for re-alignment purposes). The ability of subjects to maintain fixation was assessed outside the scanner, and in the 1.5 T scanner during the execution of the first study of D'Avossa et al [18] with the Resonance Technology infrared camera and Arlington Research software. No breaks of fixation were ever observed, either inside or outside the scanner.

### Imaging methods

Imaging data for the new data set were acquired on a 3T Philips Achieva MRI scanner, equipped with a SENSE parallel head coil (Philips, Best, Netherlands). Functional data were acquired with a single-shot gradient-echo, echo planar (EPI) sequence. Acquisition parameters were as follows: 40 axial slices, 80×80 matrix, 3 mm slice thickness, 3×3 mm in-plane voxel dimensions; 35 ms echo time (TE); 3000 ms repetition time (TR); 90°flip angle. For the old data set acquisition details are provided in reference [18]. Each scan of the main experiment comprised 126 functional volumes (the first four volumes were discarded to allow stabilization of the BOLD signal) and was repeated ten (passive fixation) or fifteen (attentive task) times. For each subject, a total of 14 scans were acquired for the motion localizers (116 functional volumes for each scan) and 6 for the retinotopy (124 functional volumes). Coverage included supra-tentorial structures and most of the cerebellum. Structural T1-weighted scans were acquired with 175 para-sagittal slices, 256×256 matrix, 1.0 mm slice thickness, 1×1 mm in-plane voxel dimensions, 8.4 ms TR, 3.9 ms TE, 8° flip angle.

On one scanning session, artifacts were noticed in the left hemisphere of the images of one subject (Sub2). A faulty connection was found in the receiver channels, which was subsequently repaired. All data were re-evaluated for image quality, and the artifact was found to be confined to one hemisphere alone, and only in this scanning session. We have included this session in the final analysis, but have masked out the hemisphere with the artefact.

We used two different software packages to analyze the data: a non-commercial software (4DFP suite and FIDL) from the NeuroImaging Laboratory at Washington University for the motion and retinotopic localizer; and Brain Voyager QX (version 1.9, Brain Innovation) for analyzing BOLD responses in the main experiment. In both experiments functional data were temporally interpolated and re-sampled to compensate for systematic slice-dependent time differences. Odd-even slice intensity differences resulting from the interleaved acquisition were eliminated. The overall image intensity was normalized within scans to a standard value to compensate for interscan intensity differences.

The data were realigned to the first volume of each scan, using a six-degree-of-freedom rigid-body affine transformation to compensate for head motion during the scanning procedure. The functional data were transformed into a standard coordinate system. The three-dimensional reconstruction of individual anatomy was obtained from averages of several high-resolution structural images. At the end, the data, both from the main experiment and from the retinotopic scans, were spatially re-sampled to a cubic voxel with a linear size of 1.0 mm [34] and analyzed using a general linear models in which the BOLD timecourse was modeled by convolving the duration of the stimulus with an assumed hemodynamic response function [35]. For each scan the independent variables also included a constant, a linear term and a set of low frequency cosine and sine functions (cutoff frequency 0.009 Hz) to remove slow varying fluctuations of the BOLD signal [36].

To generate flat representations of the cortical surface, for each hemisphere the white–gray matter junction was traced and a fiducial surface midway through the cortical surface was generated using CARET (Computerized Anatomical Reconstruction and Editing Toolkit: http://brainvis.wustl.edu/wiki/index.php/Caret:About). An automatic algorithm, supplemented by manual correction by an expert operator, removed the errors generated during the initial segmentation. This segmentation was further used to automatically generate an “explicit surface representation”, which was subsequently inflated and flattened by geometric projection.

### Stimuli and procedure

#### Main experiment design

Stimuli were generated in Matlab on a specialized graphics card, Visual Stimulus Generator (VSG 2/5, Cambridge Research Systems, run at 70 Hz), and projected by LCD projector (Sanyo, Osaka at 85 Hz) on a rear-projection screen for the new data set and on LCD goggles (Resonance Technology) equipped with infrared camera (Arlington Research Software, sample frequency 60 Hz) for the old data set. Responses were recorded using a nonferrous, fiber-optic response keypad.

**Figure 1:**
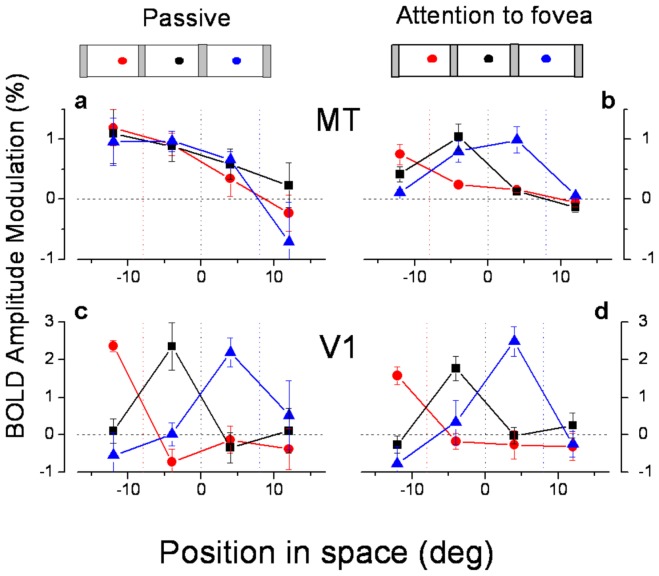
MT and V1 BOLD response amplitude. BOLD response amplitudes, averaged over subjects and hemispheres, as a function of the spatiotopic stimulus coordinates (0 is screen center), in MT (A & B) and parafoveal V1 (C & D), during passive fixation (A & C) and the foveal attentional task (B & D). The responses are color-coded by fixation (red −8°, black 0°, blue +8°: fixation indicated by the dotted colored lines). The mean responses were calculated by averaging the visual responses from homologous regions of the two hemispheres for mirror symmetric fixation directions and stimulus positions. Error bars show the between subject s.e.m., in many cases smaller than the symbol size. The responses of V1 are retinotopic, and became marginally more retinotopic in the attentional condition. In the passive viewing the responses of MT at all three fixations line up well, consistent with spatiotopic selectivity; with foveal attention they are clearly displaced in the direction of gaze, retinotopically tuned. Table S1 gives the values and significance of spatiotopic and retinotopic fits to the data.

Forty-eight randomly positioned high-contrast (0.7 Michelson) black and white dots of 9′ diameter drifted coherently (two-frame limited lifetime) either upward or downward (direction chosen at random) at 10°/s within a rectangular window of 0.8×6.5°, eliciting a strong attention-grabbing sensation of motion. Background mean luminance was about 10 cd/m^2^. The virtual windows were centered at screen locations −12°, −4°, +4° or +12° (bar and screen dimension 25% larger in the old data set) and the dots were displayed for 15 s (see inset at top of Fig. 1). Subjects maintained fixation on one of three fixation points (size 9′, contrast 0.6), positioned at either screen center (0°) or 8° left or right of it. After a variable delay of 18 s, 21 s or 24 s, the virtual window was shifted at a new location. After the stimuli had been presented once (in random order) at all four locations, the fixation point was displaced to a new location (from left to right) and a new sequence of stimulus presentations began (after a 30 s interval to allow the BOLD signal to resettle to baseline from the excitation produced by the saccadic movement to the new fixation). In each scan, all 12 conditions were presented once.

In the passive viewing condition, subjects maintained fixation, with no instructions about where to allocate attention; but given the strong salient motion it is conceivable that they directed their attention to the only stimuli presented on the display. In the attentional condition, subjects had to detect contrast decrements of the fixation point. The contrast decrement was usually around 0.5, lasted 2 frames, and was adjusted to maintain the subject's performance around 95%. The probability of the decrement was 0.5% at each frame, with a minimum separation between decrements of 400 ms.

#### Retinotopic Maps

For retinotopic mapping, the cortical representation of vertical and horizontal meridians were identified by presenting one hundred moving dots (0.33° diameter, expanding or contracting every 2.0 s, limited life time of 300 ms) in two opposing sectors (±18° degree angle) along the two principal meridians. Each sector (±18° of visual angle) extended from the screen center (corresponding to the fixation point) to the extreme border of the monitor and was presented for 15 s, twelve times in each scan. To identify the upper, lower, right and left visual quadrants, twenty two moving dots were presented within four circular sectors of ±40° angle centered along the ±45° orientation. The quadrants were presented one at the time in a clockwise order with four repetitions of each quadrant. To help localize MT+, V6 and LO, BOLD contrast for coherent versus incoherent motion responses was computed. (For details of the coherent spiral motion see ref [37]).

Paired Student's t-test was used to highlight voxels where the BOLD signal was modulated by visual stimulus position. The un-thresholded voxel-valued statistics were displayed on the flat maps [38]. Visual area boundaries were drawn by hand on the flat maps, following published conventions.

Area LO usually contained two representations of the vertical meridian probably corresponding to the LO1 and LO2 subdivisions [39], but these were not separated further here. V6 was defined as satisfying these three criteria: 1) representation of the upper visual field in the cortex, dorsal to V2/V3; 2) representation of the contralateral visual field in response to the 4 independent quadrant stimulation; 3) a strong response to flow motion [40]. The border between MT and MST was defined by the representation of the vertical meridian [41]. MST was the region responding both to ipsilateral and contralateral visual stimuli. We observed strong and reliable coherent versus incoherent motion responses within MT, MST, V6 and LO.

#### Main experiment

Both voxel-wise and ROI based analyses were performed. For each stimulus and gaze position the amplitude modulation of the response time-course was computed by subtracting the mean BOLD response for the 6 s prior to stimulus onset from the mean of the first 12 s after stimulus onset (with the response at 3 s weighted by 0.5). For each occipital cortical voxel a 4×3 stimulus response matrix (4 stimulus and 3 gaze direction) was extracted from BV in Talairach space and an affine transformation was computed to register the functional data onto the 711-2B standard atlas for Caret, the package used for visualization. BOLD responses within identified ROIs on individual subject flat maps were averaged over each hemisphere. In order to average left with right hemisphere responses, both responses and fixations of the left hemispheres were left-right flipped before averaging. For example, the BOLD responses in ROIs of the left hemisphere to stimuli at +12° and fixation −8° were averaged with BOLD responses in homologous ROIs of the right hemisphere to stimuli at −12° and fixation +8°).

### Spatiotopy Index (SI)

**Figure 2:**
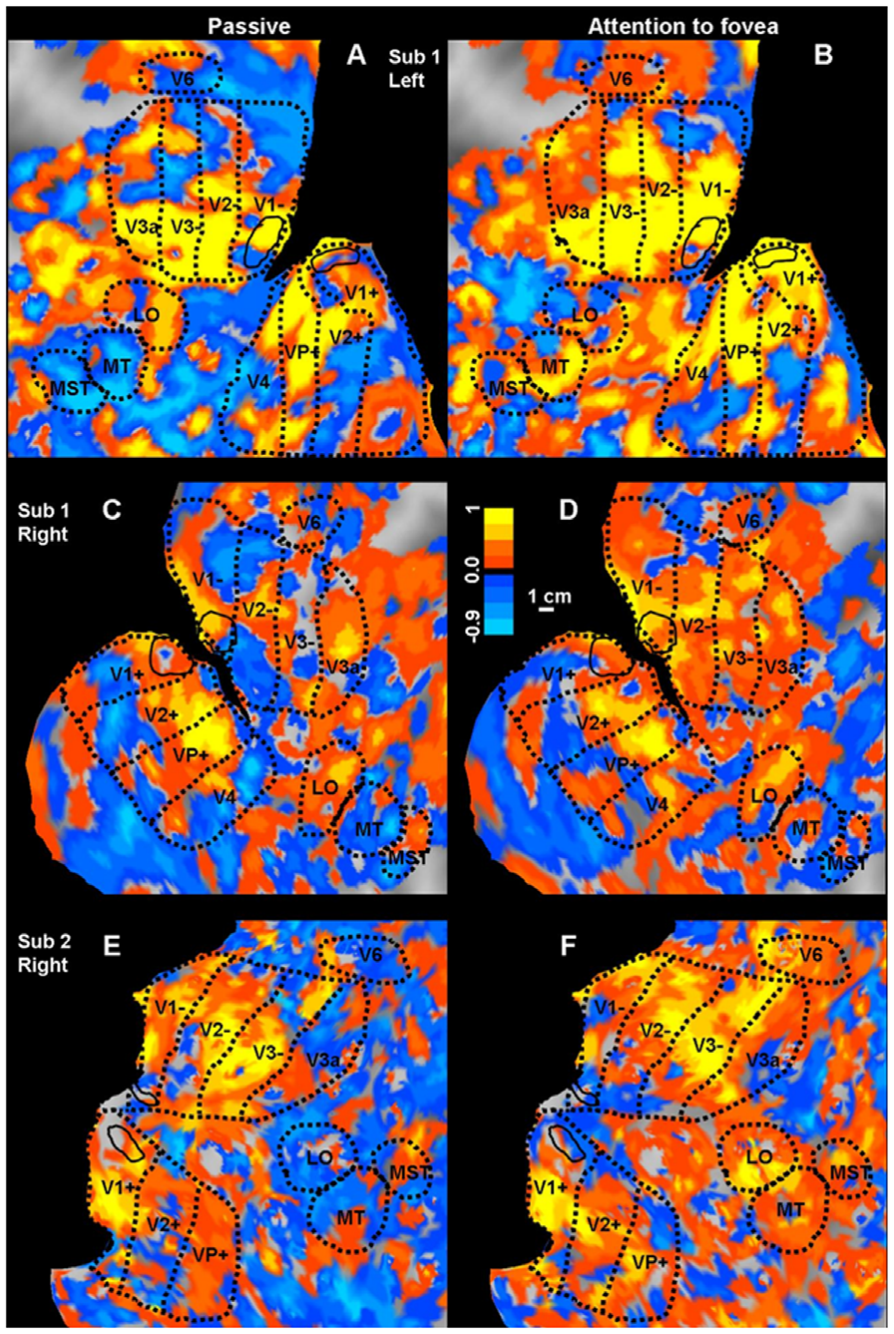
Gardner Spatiotopy Index of the visual cortex. Spatiotopy index for three representative hemispheres, the left and right hemisphere of Subject 1 (A & B, C & D) and the right of Subject 2 (E & F), for the passive-fixation condition (left: A-C-E) and attention condition (right: B-D-F). During passive fixation, there are large regions of blue (spatiotopic), particularly in dorsal cortex, including areas MT, MST, V6 and, to a lesser extent, LO. However, when performing the attention-demanding foveal task areas that were clearly spatiotopic (color-coded blue) with passive fixation, become strongly retinotopic (color-coded red/yellow) when attention is directed to the fovea. The islands within V1 inside the solid black lines indicate the parafoveal regions used for the data of Figures. 8 & 1.

For the voxel-wise analysis, we calculated a spatiotopic index (SI) similar to that used by Gardner et al [19]. Basically we calculated the summed squared difference in response amplitude for the three fixation conditions, both for a spatiotopic (screen) alignment (*resid_S_*) and a retinotopic alignment (*resid_R_*). In the spatiotopic alignment 12 comparisons are possible while for the retinotopic alignment only 8 comparisons are possible. The index SI is taken as the difference of the two average residuals divided by their sum. This is a self-normalizing index constrained between −1 (total spatiotopy) and +1 (total retinotopy).
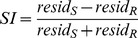
The SI index was also displayed on the flat maps (Fig. 2). The SI of voxels belonging to the various ROI between the passive and attentive condition were compared.

**Figure 3:**
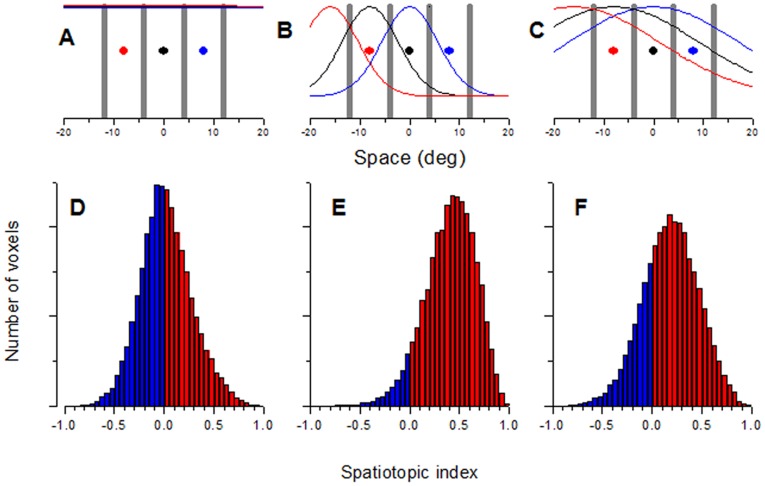
Montecarlo simulations of the spatiotopic index. Montecarlo simulations of the spatiotopic index. A–C Assumed spatial tuning of the response. Each bar represents the stimulus position and the dots the fixation; the color of the continuous curve shows the spatial selectivity tuning for each fixation. A: no tuning; B: Gaussian tuning function of 2σ = 16°, centered 8° contralaterally of fixation; C: Gaussian tuning function of 2σ = 50°, centered 8° contralaterally of fixation. D–F distribution of spatiotopic indexes, assuming that the response of each voxel is perturbed by Gaussian noise of 30% the amplitude of peak response. With no underlying selectivity, the average index is 0, with 50% spatiotopic responses, 50% retinotopic. With relatively broad selectivity, 93% of voxels were retinotopic, with only a small tail spatiotopic (E). Even 50° selectivity resulted in the majority (74%) voxels remaining retinotopic (F). No matter how unselective or noisy the responses are, they cannot produce a spatiotopic index that is consistently negative.

To determine how the spatiotopic index was affected by noise and by the size of receptive fields, we performed various Montecarlo simulations. In the first of these (Fig. 3 A&D), we assumed that the voxels had no spatial tuning but responded to all stimuli in a random fashion (Gaussian noise of 30% the amplitude). This simulation produces the distribution of spatiotopic indexes shown in Fig. 3D, centered at zero (no net spatiotopy or retinotopy). The other figures show the simulations assuming contralateral *retinotopic* tuning of the response, centered 8° contralaterally of fixation, with widths (defined as twice the Gaussian space constant: 2σ) of 16°(B) or 50° (C), with 30% Gaussian noise added to each voxel. The narrower (but still quite broad) selectivity(E) resulted in 93% of retinotopic voxels, with only a small tail spatiotopic. Even the very broad (50°-F) selectivity resulted in the majority (74%) voxels remaining retinotopic. It is important to note that no matter how large or noisy the retinotopic receptive fields are, they cannot produce a spatiotopic index that is consistently negative.

**Figure 4:**
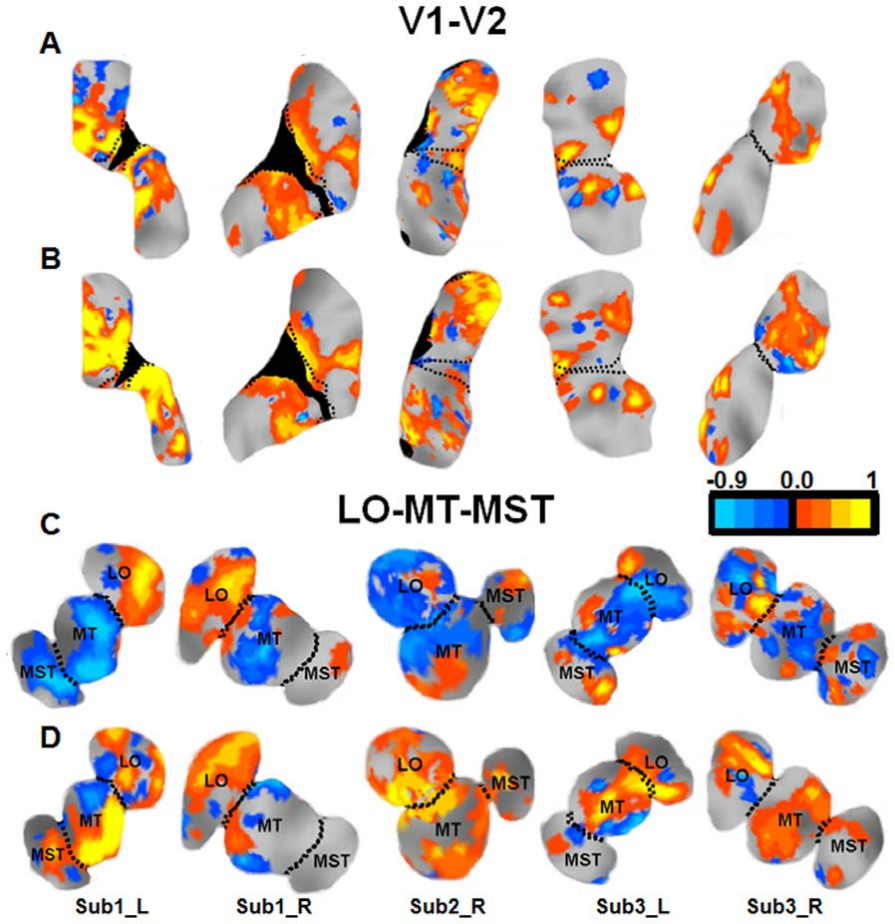
Reliable voxels for the most representative visual areas. Reliable voxels of the major visual areas considered in the spatiotopic maps showed in Fig. 2. The primary visual areas (V1 & V2- top rows) and the LO+ and MT+ complex regions (bottom rows) for each hemisphere of each subject were considered, both during the passive-fixation condition (A&C) and attention condition (B&D). Voxels were considered reliable only if the response (average for stimuli contralateral in both retinotopic and spatiotopic coordinates) correlated with the hemodynamic model (p<0.05).

**Figure 5:**
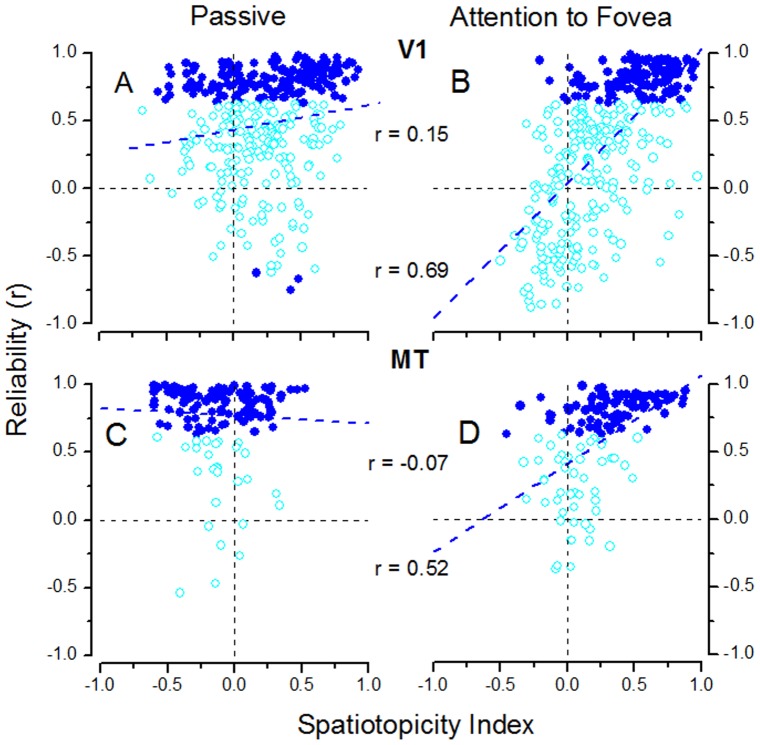
Voxel Reliability in areas V1 and MT. Reliability of voxel response plotted against spatiotopy index for the right hemisphere of subject S2. A & B area V1 (entire area); C & D area MT. Reliability was the correlation coefficient calculated by regressing the hemodynamic modeled response against the average response timecourse to all stimuli that were contralateral in both spatiotopic and retinotopic coordinates (left of both screen center and fixation). Blue symbols indicate significant reliabilities (1-tailed, p<0.05), light blue non-significant. The values of r are the correlation coefficients for regressing the reliability indexes and spatiotopy indexes.

**Figure 6:**
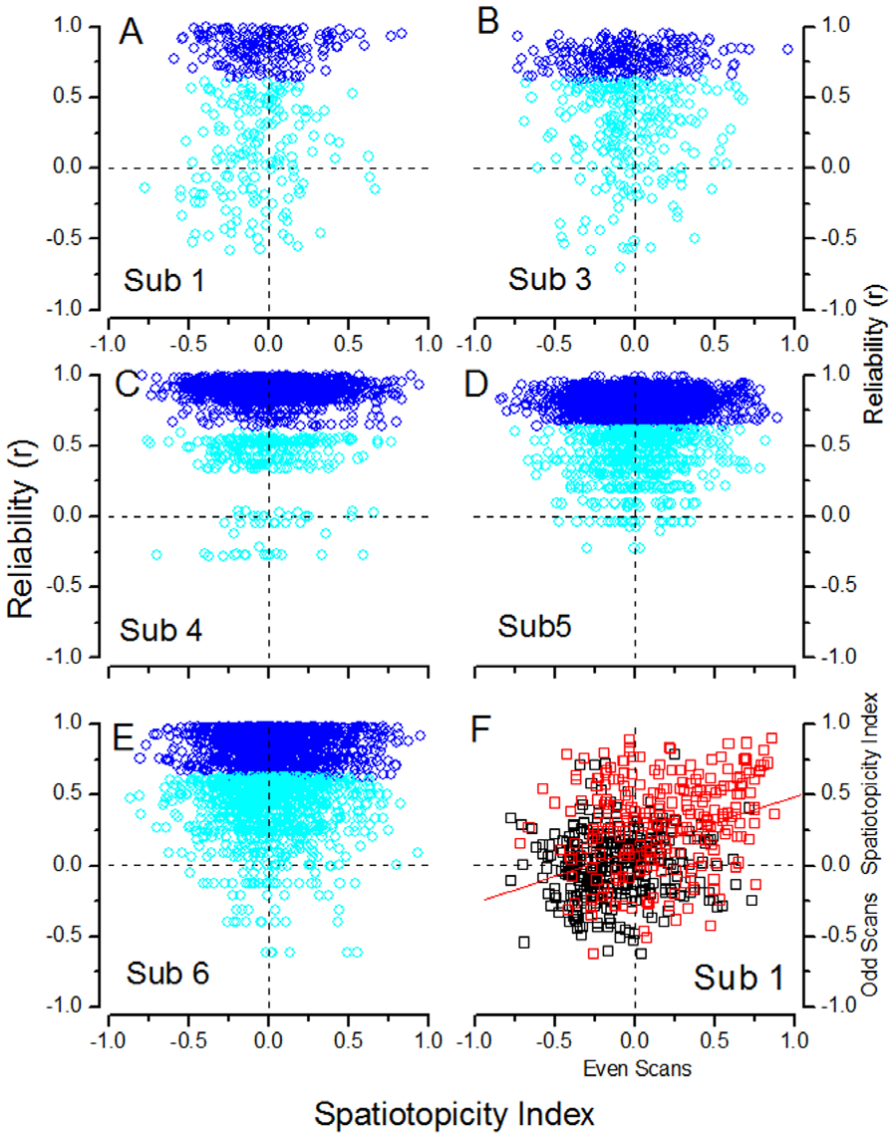
A–E Voxel Reliability in MT for all subjects. As for Fig. 5, reliability of voxel response is plotted against spatiotopy index for the other two subjects of this study (A&B) and three subjects of the previous study (d'Avossa et al [14]: C–E). Color conventions as for Fig. 5. **F** To evaluate the reliability of the Gardner spatiotopic index, the index was calculated for even runs (2, 4, 6…) and plotted against that for odd runs (1, 3, 5…) for voxels of V1 (red) and MT (black) for subject S1. The value of the correlation r = 0.42.

**Figure 7:**
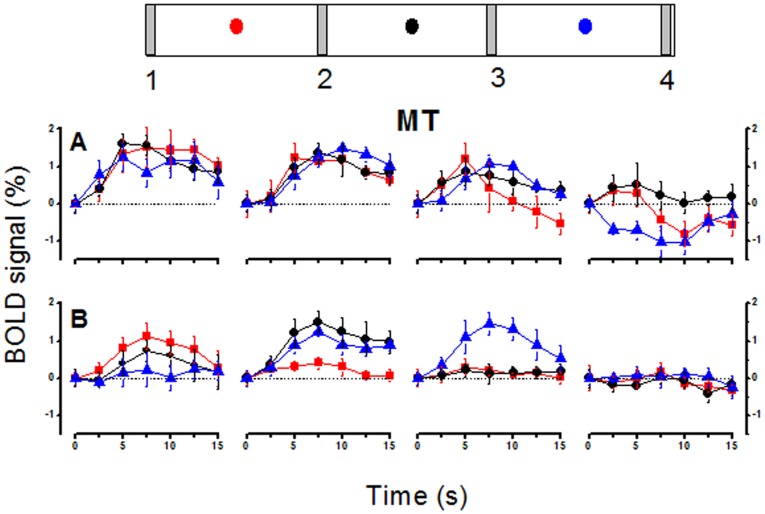
MT averaged timecourses of BOLD responses. Average timecourse of responses to four different stimuli at three different fixations, for the MT region, during passive observation (A) and attention to the fovea (B). The icons indicate the stimuli (labeled 1–4), and the dots the fixation (corresponding to the color-coded time-courses shown below). All timecourses are averaged across hemispheres whithin subjects and then across subjects, with the responses and fixations of the left hemispheres flipped to make them analogous to those of the right (see methods). Bars represent ±1 s.e.m. across subjects. The curves are shifted to have zero amplitude at time zero (stimulus onset), for display purposes.

**Figure 8:**
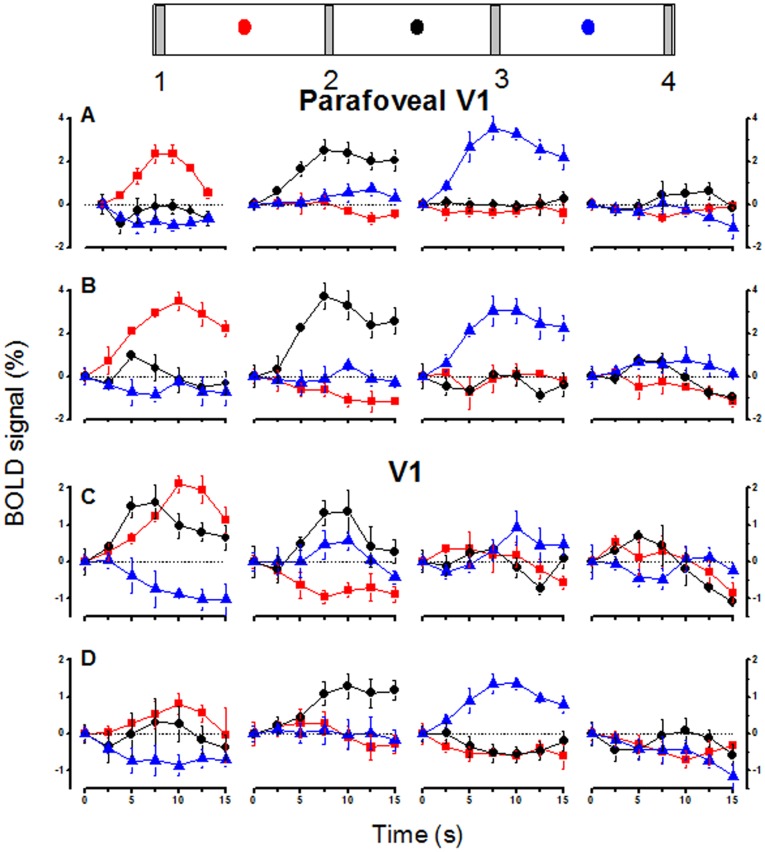
V1 averaged timecourses of BOLD responses. Average timecourse of responses to four different stimuli at three different fixations, for parafoveal V1 (a continent region where voxels responded primarily to the adjacent contralateral bar in central viewing: A and B) and the entire V1 region (C and D), during passive observation (A and C) and attention to the fovea (B and D). The icons indicate the stimuli (labeled 1–4), and the dots the fixation (corresponding to the color-coded time-courses shown below). All timecourses are averaged across hemispheres whitin subjects and then across subjects, with the responses and fixations of the left hemispheres flipped to make them analogous to those of the right (see methods). Bars represent ±1 s.e.m. across subjects. All curves are shifted to have zero amplitude time zero (stimulus onset), for display purposes.

We also calculated an index of reliability for each voxel (Figures 4, 5 and 6), by correlating the response timecourse (like those illustrated in Fig. 7 and 8 for averaged areas) with the modeled hemodynamic response.

## Results

After defining the visual areas by standard retinotopy performed with central fixation (see methods), we measured the response of the individual areas to the small motion bar presented in various screen positions for each of the three fixations. The experiment was similar to our previous study [18] and that of Gardner et al. [19]. Motion stimuli were centered in four possible positions (±4° & ±12° eccentricity) along the horizontal meridian, while subjects maintained fixation at three different points (0° & ±8°). In the passive viewing condition, subjects maintained fixation (and presumably attended to the highly salient peripheral motion at least for some of the time); in the “attentive” condition, they performed a detection task at the fovea, that was performed at about 95% correct. We measured the percentage BOLD modulation evoked by each visual stimulus at each gaze direction for all individual voxels of the whole brain.

Figure 7 shows timecourses of the evoked BOLD response in areas MT during passive fixation and when attention was directed to the fovea. The timecourses were averaged between hemispheres and then across subjects (giving the standard error estimated across subjects), after flipping both stimuli and eye position for the left hemisphere responses to make them consistent with right hemisphere response (see methods). The MT timecourses show clear spatiotopic tuning in the passive viewing condition (Fig. 7-A): the amplitude of the response to controlateral bars 1 and 2 is large, the response to bar 3 is weaker and there is no positive response to bar 4 regardless of gaze direction. Most interestingly, the pattern of responses changes when attention was allocated to the fovea, showing instead a clear pattern of retinotopic tuning (Fig. 7-B). For example bar 3, which shows a weak response during passive viewing for all gaze directions, has a strong response at fixation +8° (blue curves) in the attention condition, when it appears in the contra-lateral visual field, while the responses at other fixations nearly disappear. The responses to bar 2 also changes considerably with fixation, eliciting no ipsilateral response, showing a clear retinotopic pattern of responses. In V1 (Fig. 8), and particularly its parafoveal representation, the responses are clearly retinotopic, independently of fixation condition.

The nature of the spatial tuning of the BOLD responses is more easily observed in Fig. 1, which plots BOLD response modulation (extracted from the raw timecourses of Fig. 7 and 8: see methods) as a function of space for the three fixations. The responses at different fixations are strong in both MT and parafoveal V1 for both the attentive and passive conditions; but in MT the responses line up with each other in the passive condition (Fig. 1A), consistent with spatiotopic selectivity, while with foveal attention they are displaced in the gaze direction, consistent with retinotopic tuning (Fig. 1B). Fig. 1C&D plot the response for parafoveal V1, which shows virtually complete retinotopic selectivity in both conditions, little affected by attending to the fovea. The fact that under identical conditions (passive fixation), responses of MT are spatiotopic while those of V1 are retinotopic allows us to exclude a number of potential artifacts, such as the framing effects of the viewing goggled, or imperfect fixation since these factors should have affected equally the response of both regions.

To quantify spatiotopy vs. retinotopy we used two indexes used in previous work by d'Avossa et al [18] and by Gardner et al [19]. The first index is the shift needed to align the tuning curves obtained at the various gaze directions (minimal squared differences in responses) like those shown in Figure 1: an index of 0 implies no shift, hence perfect spatiotopy, an index of +1 a shift of the same magnitude as the fixation eccentricity, implying complete retinotopy. The Gardner index is simpler and more robust. It is based on the difference of the sum of squares of residuals for spatiotopically and retinotopically aligned responses, normalized by their sum: the index varies between −1, for purely spatiotopic responses and +1 for purely retinotopic responses (see methods and Fig. 3 for full details of index). Table S1 gives the values of these two indexes for the pooled data of Fig. 7 and 8, together with the coefficient of determination (R^2^) for the two types of fits, the proportion of variance explained by the spatiotopic model (alignment of responses in external space) and the retinotopic model (alignment of responses in retinal space), and the significance of the variance explained. Both indexes indicate very clear spatiotopy for MT during passive viewing conditions, and the coefficient of determination suggests that the spatiotopic alignment accounts for 64% of the variance. However, with attention directed to fovea, both indexes indicated reliable retinotopic behavior. Parafoveal V1 was reliably retinotopic in both conditions. For Total V1 both indexes indicate reliable retinotopy when attention was directed to the fovea but a slightly less clear behavior in the passive condition.

Since visual areas may show inhomogeneities in their responses (as Figure 8 suggests is the case for V1), it becomes important to study spatiotopy on a voxel by voxel basis, to see whether it varies within regions. We calculated for each voxel the Gardner spatiotopy index (more robust with individual voxel data), whose value was color-coded between light blue for nearly perfect spatiotopy (*SI = −0.9*) and yellow for perfect retinotopy (*SI = 0.9*). Figure 2 plots this index on a flattened representation of occipital cortex for three representative hemispheres, A, C & E for passive-fixation and B, D & F while attending to the fovea. During passive fixation, the maps show extensive regions of spatiotopic responses, particularly in dorsal cortex. Areas MT, MST, V6 and LO were mostly spatiotopic (although islands of retinotopy do exist within these regions). In the same hemispheres, performing the attention-demanding foveal task changes completely the spatial selectivity of BOLD responses to eccentric and task irrelevant motion stimuli. Areas that were spatiotopic during passive fixation became strongly retinotopic when attention was diverted to the fovea.

We also examined whether the reliability of the responses could affect the pattern of the results in the flat maps of Figure 2, by replotting the spatiotopic index only for voxels which showed a reliable BOLD response. We computed for each voxel an index of reliability [19], by correlating the predicted hemodynamic response with the timecourses of the measured response (averaged over all contralateral stimuli in both retinotopic and spatiotopic coordinates in the three fixations: 5 out of 12 stimuli-fixation conditions). We considered voxels to respond reliably if the correlation was significant (p<0.05). The spatiotopic index of these reliable voxels is shown in Figure 4, for all recorded hemispheres, for V1, LO and MT complex. It is apparent on inspection that the thresholding procedure had little effect on our findings in dorsal areas: the selectivity was primarily spatiotopic during passive viewing, changing to retinotopic when attention was diverted to the fovea. However, much of the apparent spatiotopicity of V1 and V2 voxels disappeared when considering only the reliable ones.

**Figure 9:**
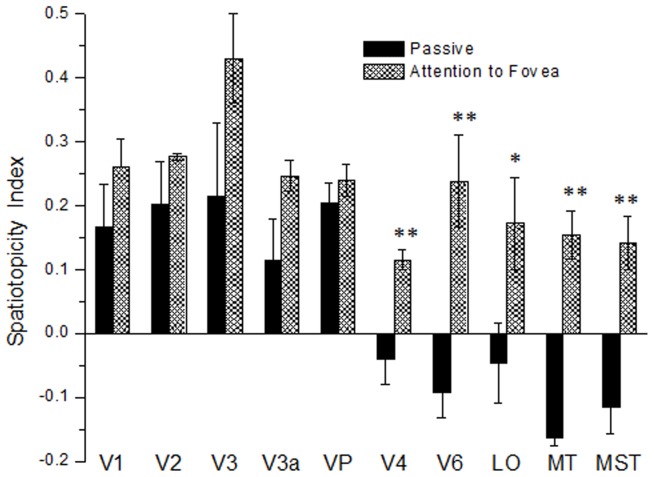
Spatiotopy Index for the most representative visual areas. Spatiotopy index for ten visual areas, calculated from individual voxels then averaged over all subjects and hemispheres, with (filled bars) and without (hatched bars) the demanding foveal task. For the regions LO-MT-MST-V6 the index clearly flips with attention, from strongly spatiotopic to strongly retinotopic. However, primary and secondary cortex, V3, V3a and VP (along the ventral stream) are retinotopic in both conditions, and attention has little influence on the index. Error bars represent the s.e.m. between subjects.

Figure 9 shows how the spatiotopic index, averaged over voxels belonging to the same functionally defined region, varies with spatial attention in ten visual areas. For regions LO, MT-MST, V6 and V4 the index flips with attention, from spatiotopic to retinotopic, this difference being statistically significant (paired, two-tailed t-test, p<0.005). However, primary and secondary cortex, V3, V3a and VP showed retinotopic responses in both conditions, with no significant change with attention. Note that the spatiotopic indexes for the retinotopic areas are around 0.2–0.3, while the indexes for the averaged response curves were much higher, around 0.8 (see Table S1). This is because the voxels responses are much noisier than the averaged responses, and noise tends to bring the value of the spatiotopic index closer to zero (Fig. 3).

**Figure 10:**
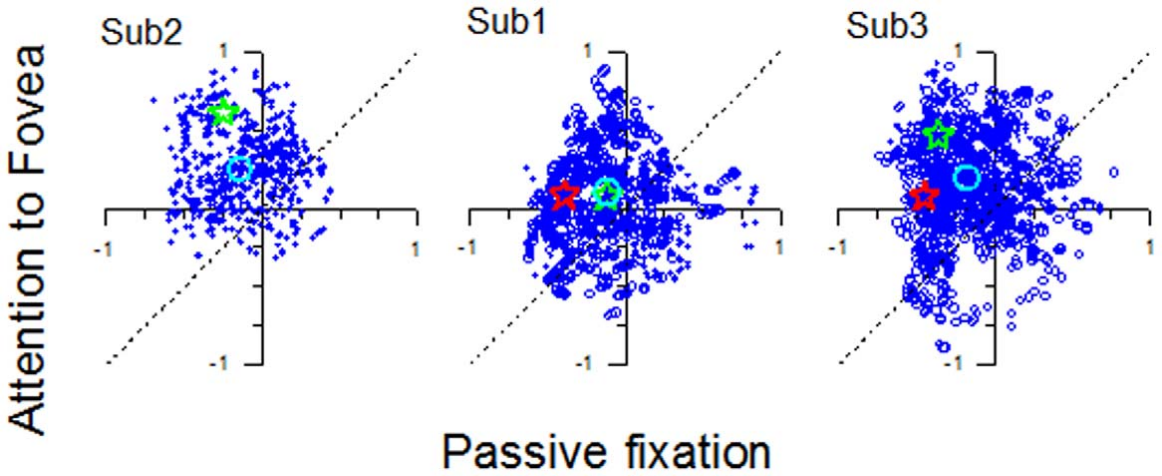
Effect of attention on the spatiotopy of MT. Scatter plot of spatiotopy indexes during the attention condition against passive fixation condition of all voxels of MT of the three subjects. An index of +1 implies that the area is perfectly retinotopic and an index of −1 implies that it is perfectly spatiotopic. The light blue open circles show the mean value of the spatiotopic index across voxels. The red and green stars are the indexes calculated on the averaged BOLD responses of all voxels in MT of the left (red) and right (green) hemisphere. The majority of voxels cluster in the upper left quadrant, spatiotopic in passive fixation and retinotopic with attention to fovea. In all three cases, the effect of attention was highly significant: paired t-tests in all conditions yielded p<0.0001.

Figure 10 illustrates the effect of attention on the spatiotopy of MT at the voxel level, and shows the voxelwise value of the spatiotopy index (and the whole region) in the attention-to-fovea condition against passive fixation. The indexes for both single voxels and the area (open stars for the two separate hemispheres) cluster in the upper left quadrant for all the three subjects, indicating that BOLD responses to motion stimuli were spatiotopic in passive fixation and retinotopic when attention was maintained at the fovea. The effect is highly significant (p<0.0001) for all hemispheres.

To account for the discrepancy between our earlier study [18] and theirs, Gardner et al [19] suggested that noisy voxels may sometimes appear to be spatiotopic simply because of random signal variations (their Fig. 5). Given the robustness of the effects reported here (see Table S1 for a summary of the significance levels of the coefficients of determination), and the fact that the effects of attention can be discerned even at the voxel level, this explanation seems highly unlikely. To demonstrate further the robustness of these reported effects, we correlated the reliability index [19] with the spatiotopy index. Fig. 5 plots, for one of the subjects (S2), the values of the reliability vs spatiotopy index, separately for data obtained during passive viewing and attention to the fovea. MT responses in the passive condition (C ) tended to be more reliable than when attention was diverted away from the stimulus (D), as may be expected [20], [21], with 84% of voxels showing significant positive correlations with the model (one-tailed test, p<0.05) in the former condition, compared with 56% in the latter. There was no tendency for the spatiotopic voxels to be less reliable than retinotopic voxels, in the passive condition, indeed reliability correlated slightly negatively with spatiotopy in area MT (r = −0.07). The fact that this dependency is very slight, together with the fact that many reliable voxels had mixed spatiotopy (near zero), suggests that this mixed tuning is a genuine phenomenon that merits further investigation. In the attention-to-fovea condition (D), the correlation was reversed (r = 0.52), suggesting that under conditions of diverted attention, the most reliable voxels in MT were the retinotopic ones, in agreement with Gardner et al. [19] findings.

Figure 5 A&B plots the reliability of voxels in area V1. Under conditions of attention to the fovea (B), the most significant voxels were retinotopic (98%), and reliability correlated strongly with retinotopy (r = 0.69). Under passive viewing (A), however, 24% of reliable voxels were spatiotopic, and the correlation with retinotopy was much lower (r = 0.15). While it is surprising that V1 shows any spatiotopy, these results agree with a recent report of Durand et al. [42] that visual responses show a gaze dependence in peripheral V1 neurons in macaque monkey. This result must nevertheless be interpreted with some caution, as the stimuli of this study were optimized to study the contentious area MT (large bars filled with fast moving stimuli), but they do open the possibility that spatiotopic selectivity may begin to occur in areas as early as V1. This possibility clearly merits further study with more optimized stimuli.

Figure 6 A–E plots the reliability against the spatiotopic index for area MT for the other two subjects of this study(A B) (S2 is shown in Fig. 5) and three from our previous study (C–E) [18], all obtained during passive viewing. In all subjects the spatiotopic voxels are as reliable as the retinotopic voxels. Therefore, the spatiotopy of BOLD response of MT is not an artifact due to poorly reliable BOLD responses. Figure 6-F tests the consistency of the spatiotopic index for one of the subject (Sub 1), by correlating the index calculated on the even scans with that of the odd scans, for areas V1 and MT. The correlation is 0.42, not unreasonable, given the susceptibility of the index to noise (Fig. 3).

### Modeling

**Figure 11:**
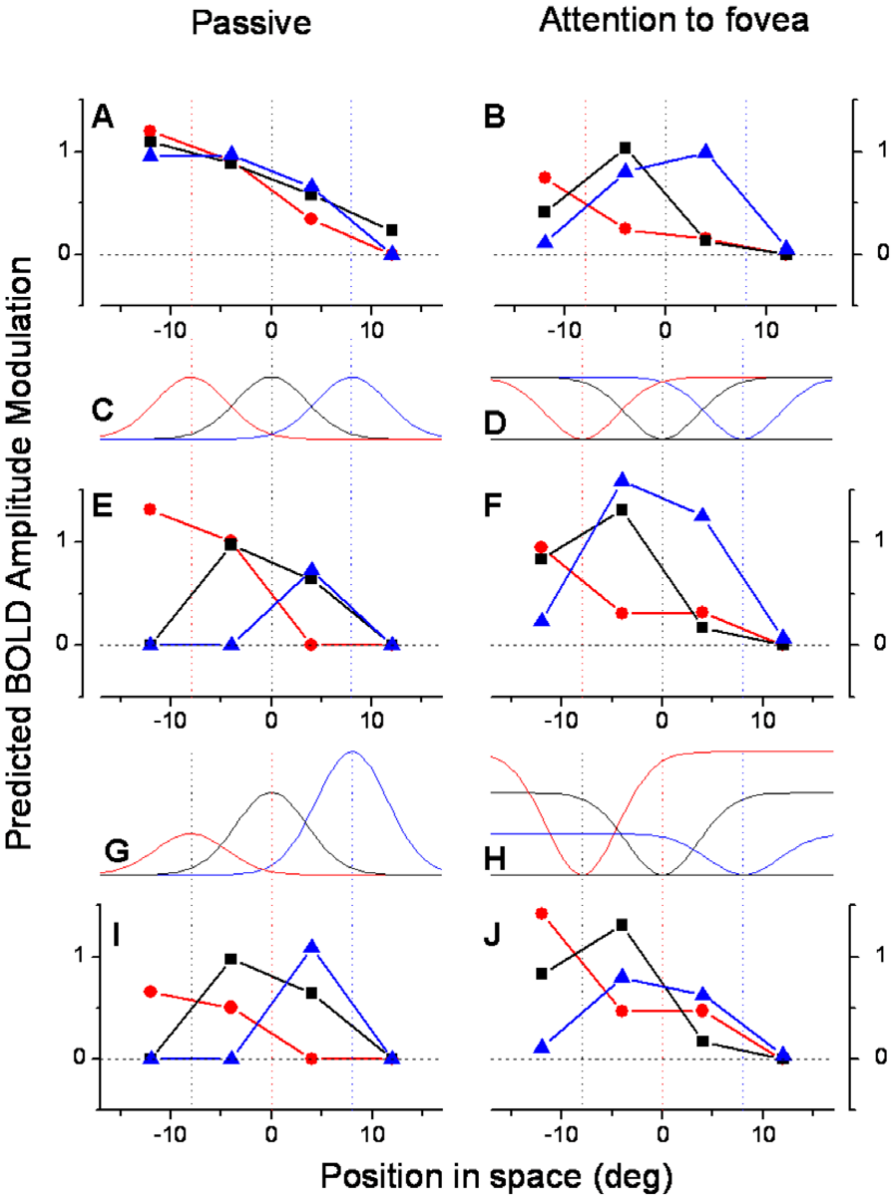
Attentional effects on MT BOLD response amplitudes. A & B: MT BOLD response amplitudes, averaged over subjects and hemispheres, as a function of the spatiotopic stimulus coordinates (0 is screen center), during passive fixation (A) and during the foveal attentional task (B). The data are taken from Fig. 1, with negative values clipped to zero (to avoid these becoming over-exaggerated during the multiplication modeling). Like Fig. 1, the responses are color-coded by fixation (red −8°, black 0°, blue +8°: fixation indicated by the dotted colored lines). C and D: The attentional effects on the BOLD response were first simulated by multiplying the non-attentional responses of Fig. 11-A with a Gaussian function with a 4° spread constant centered at fixation (C), and the attentional responses with the complementary function (1 minus the Gaussian function D). E. Boosting the response to more foveal stimuli changed the shape of the spatial tuning of BOLD responses, making them more retinotopic with D'Avossa and Gardner indexes of (0.52 and 0.34 respectively: see Table S2). F: Applying the complementary operator failed to generate spatiotopically selective responses from the retinotopic responses of Fig. 11-B, leaving both indexes of spatiotopicity virtually unchanged (Table S2). A variety of Difference of Gaussian operators were attempted, but none made the response more spatiotopic. G & H. To achieve spatiotopicity, the responses need to be modulated by a gaze-contingent function. H. shows the inverse Gaussian operators of D multiplied by a gain field that boosts leftward gaze. G shows the Gaussian boosting function multiplied by the inverse gain field (to attempt to recover the retinotopic base). I & J. Result of both attentional boost and gainfields. The spatiotopic functions of A become retinotopic (I), and the retinotopic function of B become spatiotopic (J).

Attention could affect the spatial tuning of BOLD responses in several ways. The simplest and most obvious explanation for apparent retinotopy during foveal attention is that the amplitude of BOLD responses to task irrelevant stimuli is modulated by attention. We modeled this idea by multiplying the spatiotopic responses of MT during passive viewing (Fig. 11A) with Gaussian weighting-functions centered at fixation (Fig 11C), which boost the more central response and attenuates the more peripheral ones. The results from this simulation (Fig. 11E) show that response curves become partially retinotopic, resembling more the responses with central attention (Fig. 11B). Both the d'Avossa and Gardner indexes shift towards retinotopy (Table S2). In this example the space-constant of the Gaussian was 4°, but the effect was robust over a wide range of Gaussian widths, showing that a simple boost in the response to foveal vs. peripheral stimuli can change the tuning from spatiotopic to retinotopic The effects of attention on BOLD response may account at least partly for the apparent retinotopy during the attentive condition.

On the other hand, we were unable to find a simple function (such as difference of Gaussian) that could transform the spatial selectivity obtained with central attention from retinotopic to spatiotopic. Figure 11F shows the best attempt obtained using a multiplicative model. Here the more eccentric responses (in retinal coordinates) were boosted by the function illustrated in Figure 11D, but the responses did not become spatiotopic (see Table S2). To create spatiotopy from the retinotopic responses it is necessary to consider gaze direction as well as attention. This is clear from the first experiment of d'Avossa et al, where attention was always allocated to the stimulus, but the response of MT was modulated by gaze direction: the ipsilateral response became as strong as the contralateral one when the visual stimuli were straight ahead, and gaze was to the side (Fig 7, ref [18]). Thus, boosting the central relative to peripheral retinal BOLD responses (irrespective of gaze direction) can in principle transform spatiotopic to retinotopic selectivity, but not vice versa: boosting the peripheral retinal response does not in itself transform retinotopic into spatiotopic selectivity.

Spatiotopicity cannot be generated without an interaction between the eye-position and retinal signal, and this needs to be recomputed or updated on each gaze shift. We do not here attempt a complete model of how spatiotopy may be created, but show that the most simple ‘gainfield’ concept can in principle contribute towards spatiotopy. In Figure 11J, the responses of Fig. 11F have been multiplied by a simple gradient that varies with eye position. This, together with the inverse attention function, does shift the response towards spatiotopy. Similarly, applying the inverse function to the spatiotopic response of Figure 11E makes the response far more retinotopic, showing that the function can operate in both directions. We are not suggesting that this very simple multiplication is the actual mechanism of spatiotopicity used by the visual system, but these simple simulations serve as an existence proof that gaze and attention can easily combine to convert a retinotopic response into a spatiotopic one, and vice versa.

We also point out that large receptive fields in themselves are not sufficient to create spatiotopy. In the simulation of Fig. 3C, response fields as large as 50°, extending well into the ipsilateral field, did not produce systematic spatiotopy, only noisiness.

## Discussion

The results of this study nicely reconcile the seemingly conflicting reports of d'Avossa et al. [18] and Gardner et al [19]: under passive viewing conditions (where attention was free to be directed to the stimuli) MT and many other associative areas showed a clear spatiotopic (or at least cranitopic) selectivity; but when subjects were required to perform a highly demanding foveal attentive task, the selectivity of the visually evoked BOLD response became retinotopic. The effect was most pronounced in dorsal areas, where diverting attention to the fovea changed tuning completely from spatiotopy to retinotopy; but all areas including V1 showed an increase in the retinotopic index when attention is directed at the fovea.

Evidence for spatiotopy in MT may seem at odds with physiological research, as macaque MT neurons seem to show a predominately retinotopic organization. However, there exists good neurophysiological evidence that MT receptive fields are highly plastic. For example, during memory retention of a sample-to-match motion-discrimination task, cells can be atypically modulated by stimuli in the ipsilateral visual field [43]. Eye-position signals arrive in MT [44], and these signals influence neural responsiveness via gain-field modulation [3], [45], although not obviously in a way as to create spatiotopy [5]. However, spatiotopic selectivity has been observed during pursuit eye movement in MSTl [10], [11]. And within a portion of MSTd, neurons have been described as selective for the position of visual stimuli in external 3D space (like hippocampal place cells), unchanged with body displacement or rotation [9]. This experiment does not prove that the cells are actually craniotopic, as gaze was not directly manipulated, but it does demonstrate that cells in this region can encode space in non-retinotopic coordinate systems. More recent measurements of spatial selectivity of these MSTd neurons for heading of flow motion for different gaze direction revealed no craniotopic tuning [46].

One functionally advantageous reason for non-retinotopic coding is to allow visual maps to align with those of other modalities, such as sound and vestibular signals, as clearly occurs in VIP [47], [48]. It has been shown that the presence of vestibular signals anchored in an inertial frame of reference affects the retinotopic heading tuning of neurons in MSTd [46]. This result is consistent with the general notion that spatial selectivity in MT/MST is flexible, and can adapt its reference frame for the specific task being performed. We cannot exclude the possibility that in the present study the spatiotopic tuning may emerge in combination with other sensory signals that are encoded in a craniotopic coordinates [2], such as the acoustic noise of the scanner, and attention to the fovea may interfere with this cross-sensory integration.

Even in V1 responses are modulated by gaze [49], [50], particularly peripherally tuned neurons, in a way that biases their responses towards stimuli straight ahead of the animal [42]. We too find that some V1 voxels show spatiotopic tuning in the passive viewing condition. Interestingly, BOLD responses to ipsilateral stimuli prior to saccades have been observed not only in parietal, but also in occipital human visual cortex [13], [17].

The BOLD response of many areas of parietal, temporal (including MT) and frontal cortex show a clear, spatially selective response to attention [51], [20], [21], [22], [23], [24], even in the absence of stimuli [52], [53]. The mapping of the spatial selectivity of the attentional modulation matches well the direct retinotopic input [53], although the effect has been studied only for central fixation (therefore not distinguishing between retinotopic and spatiotopic tuning). In addition, directing attention to the fovea boosts the response to stimuli near the attended target, while suppressing that to irrelevant stimuli, distant from the attended location. Our simulations (Fig. 11) show that boosting the BOLD responses of the central visual field (irrespective of gaze direction) can in principle transform spatiotopic to retinotopic selectivity, and this could account at least in part for the results, but the inverse transformation from retinotopic to spatiotopic requires an additional gaze-dependent signal. The simulations also show that a very simple gaze-related signal – a gain-field with modulation proportion to eye-position – is sufficient to create some degree of spatiotopicity. However, work has shown that the effect of attention goes beyond simply boosting the response, and can reshape and shift the receptive fields of single cells in monkey MT [25], in human MT [26] and, to a lesser extent, in human V1 [27]. The shift in peak activity can be 5–10 deg in MT (both monkey and human), probably sufficient to generate the spatial position shift needed for the spatiotopic coding.

The posterior and intra-parietal cortex comprise a distributed network of areas that control the allocation of spatial attention [54], [55], [24]. In particular, areas IPS1 and IPS2 in the intraparietal sulcus, which do not respond well to unattended stimuli, show a strong topographical organization for attentional allocation [56], [57]. The topographical organization of spatial attention signals, together with evidence of functional connectivity during sustained attention between IPS1 and IPS2 with several early visual areas [58], make these areas likely candidates for transmitting spatially specific top-down attention signals to early visual cortex [56]. There is also good deal of indirect evidence for spatiotopic selectivity for attention signals. Attention is strongly linked to motor programs [59], particularly gaze control [60], [61], which require a non-retinal map. Similarly, “inhibition of return” of attentional allocation shows primarily spatiotopic organization [28], [29], [31], although there is also good evidence for an early retinotopic component [29], [62]. A recent fMRI study showed clear evidence of both spatiotopic and retinotopic attentional enhancement of BOLD signals in early visual areas, with the spatiotopic enhancement out-lasting the retinotopic effects [30]. If, as much evidence suggests, the attentional signals of higher areas are spatiotopically tuned, then these signals – which project back to early cortical areas [58] – could be partly responsible for the spatial tuning of the BOLD responses of the early cortical areas. And if spatiotopically tuned signals arrive at relatively low-level areas of visual cortex, such as MT, these signals are almost certainly functionally important.

Further evidence for the functional importance of spatiotopy in dorsal stream areas comes from psychophysical studies. Motion signals, presumed to be processed along the dorsal stream, are integrated across saccades in a spatiotopic manner [63], [64], and motion priming occurs in spatiotopic coordinates [65]. Interestingly, motion integration depends on attention [66], although the link of attention with spatiotopy has not been demonstrated. There is also evidence that properties such as orientation and form show spatiotopic adaptation [67], all suggesting that the spatiotopy observed here is functionally important for vision.

Whatever the underlying mechanisms, the current results show a strong link between spatiotopy and attention, suggesting that mapping objects into a spatiotopic frame requires attention. Why should only attended objects show spatiotopy? Most researchers now believe that the visual system does not construct detailed spatiotopic maps of the entire image, but that only salient features are transferred from one fixation to another [68], [69]. Cavanagh and colleagues [32] have gone a step further and proposed that information transfer across saccades is based on a system of “attentional pointers”, which are updated on each eye-movement. These pointers are linked to identity information and serve to establish a workable visual architecture of the external scene. This general idea is consistent with our findings that only attended stimuli show spatiotopic tuning.

## Supporting Information

Table S1
**Spatiotopy and retinotopy indexes for MT and V1.** The table gives the values of *d'Avossa index* [14] where 0 indicates full spatiotopy and 1 full retinotopy – and the *Gardner index* [24] where −1 indicates full spatiotopy and +1 full retinotopy, for the various conditions of Fig. 1. *R^2^* is the *coefficient of determination* for the perfect retinotopic or spatiotopic alignment of responses. A value of 1 means that the model accounts for all the variance, a value less than zero means that the model is worse than the mean in explaining the variance. Where SS means sums of squares, *R^2^* is given by:
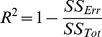
The statistical significance of the coefficient of determination (compared with the mean) was calculated by t-test with *n*-2 degrees of freedom. For the spatiotopic alignment, n = 12, for the retinotopic alignment, n = 10. The double stars refer to p<0.01. Obviously, if the explained variance is significantly different from the mean, it is significantly different from a negative value (worse than the mean).
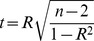

(DOC)

Table S2 **Simulations of MT responses with attentional boost and gaze-dependent gain fields.** Same indexes and significance testing as for Table S1, calculated for the simulations described in the text. The data are taken from Fig. 1, with negative values clipped to zero.
(DOC)
