# Supplementary material for: Spatiotopic Coding of BOLD Signal in Human Visual Cortex Depends on Spatial Attention
Source: PLoS One. 2011 Jul 7;6(7):e21661. doi: 10.1371/journal.pone.0021661 (PMC3131281; doi:10.1371/journal.pone.0021661)
Supplement: Table S1 — Spatiotopy and retinotopy indexes for MT and V1. The table gives the values of d'Avossa index [14] where 0 indicates full spatiotopy and 1 full retinotopy – and the Gardner index [24] where −1 indicates full spatiotopy and +1 full retinotopy, for the various conditions of Fig. 1. R2 is the coefficient of determination for the perfect retinotopic or spatiotopic alignment of responses. A value of 1 means that the model accounts for all the variance, a value less than zero means that the model is worse than the mean in explaining the variance. Where SS means sums of squares, R2 is given by:The statistical significance of the coefficient of determination (compared with the mean) was calculated by t-test with n-2 degrees of freedom. For the spatiotopic alignment, n = 12, for the retinotopic alignment, n = 10. The double stars refer to p<0.01. Obviously, if the explained variance is significantly different from the mean, it is significantly different from a negative value (worse than the mean). (DOC) [file pone.0021661.s001.doc]

**Table 1**

|  | d’Avossa index | Gardner index | R2 (retino) | R2 (spatio) |
| --- | --- | --- | --- | --- |
| Parafoveal V1 passive | 1.02 | 0.83 | 0.79** | –1.64 |
| Parafoveal V1 attention | 0.84 | 0.81 | 0.76** | –1.41 |
| MT passive | –0.02 | –0.64 | –0.80 | 0.64** |
| MT attention | 0.81 | 0.65 | 0.69** | –0.50 |
| All V1 passive | 0.56 | 0.41 | 0.07 | –1.34 |
| All V1 attention | 0.71 | 0.67 | 0.58** | –1.18 |
