# Supplementary material for: Spatiotopic Coding of BOLD Signal in Human Visual Cortex Depends on Spatial Attention
Source: PLoS One. 2011 Jul 7;6(7):e21661. doi: 10.1371/journal.pone.0021661 (PMC3131281; doi:10.1371/journal.pone.0021661)
Supplement: Table S2 — Simulations of MT responses with attentional boost and gaze-dependent gain fields. Same indexes and significance testing as for Table S1, calculated for the simulations described in the text. The data are taken from Fig. 1, with negative values clipped to zero. (DOC) [file pone.0021661.s002.doc]

**Table 2**

|  | d’Avossa index | Gardner index | *R^2^* (retino) | *R^2^* (spatio) |
| --- | --- | --- | --- | --- |
| **Passive** (clipped positive) | 0.17 | –0.75 | –0.46 | 0.83** |
| Foveal attentional boost | 0.52 | 0.34 | -0.02 | -1.07 |
| Boost + inverse gainfield | 0.63 | 0.47 | 0.32* | -0.88 |
|  |  |  |  |  |
| **Attentive** (clipped positive) | 0.80 | 0.69 | 0.72** | –0.63 |
| Inverse foveal boost | 0.84 | 0.58 | 0.59** | -0.46 |
| Inverse boost + gainfield | 0.34 | 0.24 | 0.21 | −0.36 |
